# Effect of inter- row spacing and seed rate on growth; Yield and yield components of fenugreek (*Trigonella foenum - graecum* L.) in Tehuledere district, Northeastern Ethiopia

**DOI:** 10.1371/journal.pone.0355598

**Published:** 2026-08-14

**Authors:** Maria Tesfaye Gebeyehu, Derbew Belew Yohannes, Tilahun Tadesse Feleke, Zenebe Gebremedhin Berhe

**Affiliations:** 1 College of Agriculture and Environmental Sciences, Department of Plant Sciences, University of Gondar, Gondar, Ethiopia; 2 College of Agriculture and Veterinary Medicine, Department of Horticulture and Plant Sciences, Jimma University, Jimma, Ethiopia; 3 Ethiopian Institutes of Agricultural Research, Fogera National Rice Research and Training Center, Ethiopia; 4 College of Agriculture and Environmental Sciences, Department of Plant Sciences, University of Gondar, Gondar, Ethiopia; Tokyo University of Agriculture, JAPAN

## Abstract

Fenugreek (*Trigonella foenum-graecum L.*) is well-adapted for growing in northeastern Ethiopia due to its ideal agro-climatic conditions. Despite its potential, limited research has been conducted in Ethiopia to improve the productivity of this orphan crop particularly regarding optimal row spacing and seed rate. The current study evaluated the effects of inter-row spacing and seed rate on growth, yield components, and grain yield of fenugreek. Field experiments were conducted from July 2022 to January 2023 at Kete and Korke kebeles in the Tehuledere district of northeastern Ethiopia. The experiment was conducted using a randomized complete block design with a factorial arrangement of five seed rates (15, 20, 25, 30, and 35 kg ha ⁻ ¹) and three row spacings (20, 30, and 40 cm) with three replications. Statistix 10.0 software was used to evaluate data on growth and yield components. The highest seed yield (0.98 t ha ⁻ ¹) was found at 20 cm row spacing with a seed rate of 30 kg ha ⁻ ¹, while the lowest yield (0.36 t ha ⁻ ¹) was found at 40 cm spacing with a seed rate of 15 kg ha ⁻ ¹, according to the results. To maximize fenugreek yield in the Tehuledere district and other similar agroecological areas, a row spacing of 20 cm and a seed rate of 30 kg ha ⁻ ¹are suggested. Further multi-season and multi-location studies are needed to validate the recommended seed rate and row spacing across varying agroecological conditions.

## Introduction

Fenugreek (*Trigonella foenum-graecum* L.) is an annual, self-pollinated diploid legume (2n = 16) belonging to the family Fabaceae [1]. It is commonly grown in China, India, Egypt, Ethiopia, Morocco, Ukraine, Greece, and Turkey [2]. It originated in the nations that border the eastern shores of the Mediterranean [3]. In Ethiopia, fenugreek is mainly cultivated in highland areas between 1800 and 2300 meters above sea level, where the climate features alternating wet and dry seasons [4].

The crop has multiple uses, including as a food source, spice, medicinal plant, livestock feed, green manure, and rotational crop due to its nitrogen-fixing capacity [5; 6]. It is one of the main seed spices grown in Ethiopia's Amhara region, and smallholder farmers and private investors around the nation are eager to produce it [7].

Among agronomic management practices, seed rate and row spacing play a critical role in determining crop performance, as they directly influence plant population and resource utilization. Achieving an optimal plant density is essential for maximizing growth and yield. However, limited research has been conducted on fenugreek crop spacing and seed rate. Therefore, by implementing better management techniques coupled with the ideal seed rate and spacing, there is a significant chance of raising this commercial crop's output. Optimal seed rate and row spacing combinations vary by site and lack a universal standard. Reported recommendations range from wide (40 cm), intermediate (30 cm), and narrow (20 cm) spacings, often combined with varying seed rates, depending on local agroecological conditions [8–11].

The novelty of this study lies in evaluating the interactive effects of different inter-row spacing and seed rates under the specific agroecological conditions of Tehuledere District, where such evidence is currently lacking. By addressing this gap, the study provides evidence-based guidance to optimize fenugreek productivity and strengthen decision-making for farmers and extension services in northeastern Ethiopia.

Fenugreek is the sixth most- produced highland pulse and spice crop in Ethiopia, with around 50,742.23 tons of fenugreek seed produced annually on approximately 42,344.28 hectares of agricultural land [12]. The nation's fenugreek production was 1.198 tons ha^-1^, which is far less than its potential yield of 2.3 tons ha^-1^. Fenugreek productivity was 1.34 tons ha^-1^ in the Amhara region and 0.749 tons ha^-1^ in the South Wollo zone [12,13]. Enhancing fenugreek yield has received little attention, despite the crop's significance and potential. The effects of plant spacing and seed rate on fenugreek yield and yield components in Ethiopia and the study area are poorly understood. Therefore, this study was conducted to test the following hypothesis: Inter-row spacing and seed rate significantly affect growth, yield components, and yield of fenugreek. There exists an optimal combination of seed rate and row spacing that maximizes fenugreek productivity under local conditions. For fenugreek production under particular local conditions, it is crucial to determine the proper seed rate and row spacing. The present study was undertaken to evaluate the effects of different inter-row spacing and seed rates on the growth, yield components, and overall yield of fenugreek in the Tehuledere District.

## 2. Materials and methods

### 2.1. Description of the study area

The field experiment was conducted during the 2022/2023 main cropping season (July 2022 to January 2023) at two representative fenugreek-growing locations, Kete and Korke, in the Tehuledere District, South Wollo Zone, Amhara National Regional State, northeastern Ethiopia. The Kete experimental site is located at 11°14′N and 39°40′E at an altitude of approximately 1900 m above sea level (m.a.s.l.). The site receives a mean annual rainfall of 1204.6 mm, with average daily maximum and minimum temperatures of 28.2°C and 6.6°C, respectively [14]. The Korke site is situated at an altitude of approximately 2200 m.a.s.l., with average daily maximum and minimum temperatures of 24°C and 5°C, respectively [14]. The experimental site's soil has a sandy loam texture. The region is well-known for its fenugreek production.

### 2.2. Experimental design and treatments

The experiment was carried out under rain-fed conditions at farmer training centers in Kete and Korke with permission from local agricultural authorities and landholders during the main cropping season. The fenugreek variety used in this study was ‘Woreilu’, which is widely grown in the area due to its high yield potential and desirable oleoresin content. The seeds are small, hard, and yellowish-brown.

The treatments consisted of a factorial combination of three inter-row spacing (20 cm, 30 cm, and 40 cm) and five seed rates (15, 20, 25, 30, and 35 kg ha ⁻ ¹). The treatment combination representing the locally recommended practice was used as a reference point for comparison. The experiment was arranged in a randomized complete block design (RCBD) with three replications. A uniform application of 100 kg ha ⁻ ¹ of NPS fertilizer was applied to all plots at sowing [15].

### 2.3. Plot layout and experimental units

Each experimental plot had a gross size of 3.6 m × 1.5 m (5.4 m^2^). Depending on the row spacing treatments, plots contained 18, 12, or 9 rows for 20 cm, 30 cm, and 40 cm spacing, respectively. Seeds were sown by drilling along the rows.

To minimize border effects, one row on each side of the plot was excluded, and an additional row was designated for destructive sampling. The remaining central rows were used for data collection. A spacing of 0.5 m between plots and 1 m between blocks was maintained throughout the experiment.

### 2.4. Cultural practices

Before sowing, the soil was prepared using an oxen-driven local plow (Maresha) in accordance with traditional plowing methods. A field pattern was created, the area was plowed three times, and the final plowing was used for sowing in compliance with the design parameters. Each treatment was then randomly assigned to experimental plots. Every crucial agronomic technique, including cultivation, harvesting, and weeding, was used in accordance with the advice.

### 2.5. Data collection

Data were collected on various growth, yield components, and yield parameters as described below:

Days to 50% flowering: Number of days from emergence until 50% of plants in a plot had open flowers, determined by visual observation.

Days to 90% maturity: Number of days from emergence until approximately 90% of plants reached physiological maturity, indicated by yellowing of leaves and pods.

Plant height (cm): Measured at physiological maturity from the base to the tip of ten randomly selected plants per plot, and the average value was recorded.

Number of primary branches per plant: Counted from ten randomly selected plants at maturity and averaged.

Total number of nodules: Five representative plants were randomly sampled from the destructive sampling row at mid-flowering (50% flowering stage). Plants were carefully excavated to recover the entire root system, which was gently washed to remove adhering soil. The total number of nodules per plant was counted manually to quantify nodulation. The functional activity of nodules was assessed visually by examining internal nodule pigmentation; nodules exhibiting pink to reddish coloration due to leghemoglobin were considered active and indicative of effective nitrogen fixation.

Number of pods per plant: Determined by counting pods from ten randomly selected plants at harvest and calculating the mean.

Number of seeds per pod: Seeds obtained from pods of sampled plants were counted and divided by the number of pods to obtain an average.

Thousand seed weight (g): Thousand-seed weight was determined by counting and weighing 1000 seeds from each plot using an electronic balance. Prior to weighing, seed samples were air-dried to a uniform moisture content (approximately 10–12%) to minimize the effect of seed moisture on weight measurements.

Biomass yield (kg ha ⁻ ¹): Total above-ground biomass (including leaves, stems, and seeds) was harvested from the net plot area, air-dried to constant weight, and converted to kilograms per hectare.

Seed yield (kg ha ⁻ ¹): Seeds harvested from the net plot area were cleaned, air-dried to approximately 10–12% moisture content before weighing, and the yield was converted to kilograms per hectare.

Harvest index: Calculated as the ratio of seed yield to total above-ground biomass yield.

### 2.6. Statistical data analysis

Data were subjected to analysis of variance (ANOVA) using Statistix 10.0 software. The experiment followed a factorial arrangement in a randomized complete block design (RCBD) with three replications. For combined analysis across locations, location was considered as a random effect, while seed rate and row spacing were treated as fixed effects. The interaction effects among factors were also tested. Mean separation was performed using the Least Significant Difference (LSD) test at the 5% probability level. Prior to analysis, assumptions of ANOVA were checked. Normality of residuals was tested using the Shapiro–Wilk test, while homogeneity of variance was assessed using Hartley’s F-max test. Where assumptions were met, a combined analysis was conducted.

## 3. Results

### 3.1. Growth and phenological parameters as influenced by seed rate and inter-row Spacing

#### Effect on plant height.

The combined analysis across locations showed that the interaction between seed rate and inter-row spacing did not significantly affect plant height. However, the main effects of inter-row spacing and seed rate were significant (P ≤ 0.05). For inter-row spacing, plant height varied from 46.96 to 49.70 cm. The tallest plants (49.70 cm) were observed at 40 cm inter-row spacing, while the shortest plants (46.96 cm) were recorded at 30 cm inter-row spacing. Seed rate also significantly influenced plant height. Plant height ranged from 45.07 cm to 49.80 cm, with the tallest plants (49.80 cm) occurring at the highest seed rate of 35 kg ha ⁻ ¹ and the shortest (45.07 cm) at the lowest seed rate of 15 kg ha ⁻ ¹.

#### Effect on number of branches per plant.

The number of branches per plant was significantly affected by the interaction between seed rate and row spacing (P ≤ 0.001) (Table 1). The highest number of branches per plant (14.0) was recorded at 40 cm inter-row spacing combined with 20 kg ha ⁻ ¹ seed rate, whereas the lowest value (8.9) occurred at 30 cm inter-row spacing with 15 kg ha ⁻ ¹ seed rate. Location also significantly affected the number of branches, with Kete producing more (12.16) than Korke (11.50). Wider inter-row spacing (40 cm) significantly increased branch number compared with narrower spacing, while the lowest seed rate (15 kg ha ⁻ ¹) produced fewer branches than the higher seed rates.

**Table 1:** Interaction effects of seed rate and inter-row spacing on growth and phenological parameters of fenugreek.

Treatments		Plant height (cm)	No. of branches / plant	Days to 50% flowering	Days to 90% maturity
Inter-Row Spacing (cm)	Seed rate (Kg ^ha-1^)				
20	15	47.4^abcd^	10.9^f^	55.3^cde^	118.6^f^
	20	48.1^abcd^	10.7^f^	55.6^bcde^	122.6^ef^
	25	46.1 cd	12.1^cde^	58^abcd^	123.5^e^
	30	48.1^abcd^	10.6^f^	58.6^ab^	128.5^d^
	35	48.2^abcd^	11.1^ef^	60.6^a^	129.6 cd
30	15	43.9^d^	8.9^g^	54.5^e^	128.8 cd
	20	45.9 cd	11.6^def^	56.6^bcde^	129.3 cd
	25	48.3^abcd^	11.67^cdef^	57.1^bcde^	129.6 cd
	30	47.3^bcd^	12.3 cd	58.5^ab^	131.8^bcd^
	35	49.2^abc^	12.8abc	58.1abcd	133.3abc
40	15	43.9^d^	13.7^ab^	55.1^de^	132.3^bcd^
	20	50.7^abc^	14^a^	57^bcde^	131.6^bcd^
	25	52.3^a^	12.8^ab^	57.6^abcd^	135^ab^
	30	49.8^abc^	12.7^bcd^	57.5^bcde^	136.1^ab^
	35	52^ab^	11.7^cdef^	58.3^abc^	137^a^
Mean		48.1	11.85	57.26	129.8
Standard Error		2.45	0.57	1.53	2.3
CV (%)		8.85	8.43	4.64	3.08
LSD		4.9	1.15	3.07	4.62

Means followed by the same letter within a column are not significantly different at p ≤ 0.05 according to LSD test.

#### Effect on days to 50% flowering.

The interaction between seed rate and row spacing across locations did not significantly affect days to 50% flowering. However, the main effect of seed rate was significant for days to 50% flowering. The longest duration to reach 50% flowering (59 days) occurred at the highest seed rate of 35 kg ha ⁻ ¹, whereas the shortest duration (55 days) was recorded at the lowest seed rate of 15 kg ha ⁻ ¹.

#### Effect on 90% physiological maturity.

The main effects of location, inter-row spacing, and seed rate significantly (P ≤ 0.001) influenced the days to 90% physiological maturity. The longest maturity duration (132.56 days) was observed at Kete Kebele, while the shortest period (127.2 days) occurred at Korke Kebele. Inter-row spacing also had a significant effect on maturity. The longest maturity duration (134.4 days) was recorded at 40 cm inter-row spacing, whereas the shortest duration (124.6 days) occurred at 20 cm inter-row spacing. Seed rate similarly influenced maturity, with the highest value (133.3 days) at 35 kg ha ⁻ ¹ and the lowest (126.6 days) at 15 kg ha ⁻ ¹.

### 3.2. Nodulation, yield components and yield parameters as affected by seed rate and inter-row spacing of fenugreek

#### Effect on the number of nodules per plant.

The interaction between seed rate and inter-row spacing significantly affected nodulation (P ≤ 0.001). The highest number of nodules (16.3) was obtained from 20 cm row spacing with 30 kg ha ⁻ ¹ seed rate, whereas the lowest (9.3) occurred at 20 cm spacing with 15 kg ha ⁻ ¹. Location, inter-row spacing, and seed rate also had significant independent effects, with Kete, 20 cm spacing and 30 kg ha ⁻ ¹ producing the highest mean number of nodules per plant (Table 2).

**Table 2:** Interaction effects of inter-row spacing, seed rate and location on nodulation and yield components of fenugreek.

Parameter	Number of nodule per plant	Number of pods per plant	Number of seeds per pod
Location	0.01	0.000	0.002
Spacing	0.003	0.000	0.000
Seed rate	0.0000	0.000	0.000
Location* Spacing	0.013	0.378	0.235
Location* seed rate	0.145	0.11	0.169
Spacing*seed rate	0.000	0.000	0.000
Location*spacing*seed rate	0.0002	0.02	0.01
Error MS	0.96	0.125	0.164
Mean	12.65	37.68	16.75
CV	7.76	0.94	2.42

#### Effect on number of pods per plant and number of seeds per pod.

The interaction between inter-row spacing and seed rate significantly affected both the number of pods per plant and the number of seeds per pod (P ≤ 0.001). The highest pod number (39.6) and seed number per pod (20.4) were obtained from 20 cm inter-row spacing combined with 30 kg ha ⁻ ¹ seed rate, whereas the lowest values were recorded at 20 cm inter-row spacing with 15 kg ha ⁻ ¹ seed rate and 40 cm inter-row spacing with 20 kg ha ⁻ ¹ seed rate, respectively (Table 3). In addition to the significant interaction effects, location, inter-row spacing, and seed rate independently influenced the number of pods per plant (P ≤ 0.001). The highest mean number of pods per plant was recorded at 40 cm inter-row spacing, whereas the lowest occurred at 30 cm inter-row spacing. Seed rates of 20–35 kg ha ⁻ ¹ produced statistically similar number of pod per plant, while 15 kg ha ⁻ ¹ resulted in the lowest value.

**Table 3:** Interaction effects of seed rate and inter-row spacing on nodulation and yield components.

Treatments		Number of nodules per plant	Number of pods per plant	Number of seeds per pod
Inter-Row Spacing (cm)	Seed rate (Kg ^ha-1^)			
20	15	9.3^g^	35.5^j^	16^fg^
	20	10.83^f^	36.7^h^	16.7^e^
	25	13.9^bcd^	38.9^b^	18.6^b^
	30	16.3^a^	39.6^a^	20.4^a^
	35	14.5^bc^	39.3^ab^	18.8^b^
30	15	11.2^f^	36.3^i^	15.6^g^
	20	12.86^de^	38.2^c^	16.8^de^
	25	12.9^de^	38.2^c^	17.3 cd
	30	10.8^f^	38.1 cd	17.1^cde^
	35	12.8^de^	38.4^c^	17.4^c^
40	15	10.9^f^	36^i^	16.2^f^
	20	12.4^e^	37.1^gh^	14.1^i^
	25	13.4^cde^	37.2^fg^	14.5^i^
	30	15^b^	37.5^ef^	15^h^
	35	12.43^e^	37.7^de^	16.2^f^
Mean		12.65	37.68	16.75
Standard Error		0.56	0.2	0.23
CV (%)		7.76	0.94	2.42
LSD		1.13	0.4	0.46

Means followed by the same letter within a column are not significantly different at p ≤ 0.05 according to LSD test.

#### Effect on thousand-seed weight and grain yield.

Thousand-seed weight was significantly (P ≤ 0.05) affected by the interaction effect of seed rate and inter-row spacing across locations. The combination of 20 cm inter-row spacing and a seed rate of 30 kg ha ⁻ ¹ in Korke Kebele produced the highest thousand-seed weight of (22.1 g), which was on par with that obtained in Kete Kebele. Conversely, the combination of a 40 cm row spacing and a seed rate of 15 kg ha ^−1^ in Kete Kebele yielded the lowest thousand-seed weight (14.8 g).

The interaction between seed rate and inter-row spacing had a significant effect on thousand- seed weight, which ranged from 15.14 g to 21.19 g. Plants seeded at 20 cm inter-row spacing with a seed rate of 30 kg ha ⁻ ¹ had the highest thousand-seed weight (21.19 g), while plants sown at wider inter-row spacing of 30 cm and 40 cm combined with a seed rate of 15 kg ha ⁻ ¹ had the lowest thousand-seed weight (15.14 g) (Table 4).

**Table 4:** Interaction effects of seed rate and inter-row spacing on yield components and yield of fenugreek.

Treatments		Thousand- seed weight (g)	Grain yield (t ha^-1^)	Biological yield(t ha^-1^)	Harvest index(%)
Spacing (cm)	Seed rate (kg ha^-1^)				
20	15	15.9 cd	0.51^h^	4.28^i^	11.93^de^
	20	17.9^b^	0.59^fg^	4.61^h^	12.87 cd
	25	17.1b^c^	0.81^c^	6.26^c^	12.98 cd
	30	21.1^a^	0.98^a^	6.7^a^	14.75^a^
	35	17.9^b^	0.83b	6.46^b^	12.96 cd
30	15	15.1^d^	0.47^i^	3.93^j^	12.18^cde^
	20	18.3^b^	0.6^f^	4.5^h^	14.84^a^
	25	18.4^b^	0.7^e^	4.98^g^	14.11^ab^
	30	18.2^b^	0.76^d^	5.3ef	14.45^a^
	35	17.57^b^	0.75^d^	5.18e^f^	14.45^a^
40	15	15.15^d^	0.36^j^	3.48^k^	10.41^f^
	20	18.3^b^	0.58^g^	5.18^f^	11.18^ef^
	25	18.1^b^	0.69^e^	5.36^e^	12.86 cd
	30	17.5^b^	0.7^e^	5.63^d^	12.51 cd
	35	17.6^b^	0.7^e^	5.43^e^	13b^c^
Mean		17.64	0.67	5.15	13.04
Standard Error		0.69	0.3	0.07	0.52
CV (%)		6.79	2.45	2.55	7.03
LSD (5%)		1.38	0.01	0.15	1.05

Means followed by the same letter within a column are not significantly different at p ≤ 0.05 according to LSD test.

Thousand-seed weight had a significant (P ≤ 0.001) effect on location. Korke kebele had the highest thousand-seed weight (18.2g), whereas Kete kebele had the lowest thousand-seed weight (17g). Thousand-seed weight was not greatly affected by inter- row spacing. Thousand- seed weight was significantly (P ≤ 0.001) influenced by seed rate. The seed rate of 30 kg ha ⁻ ¹ produced the highest thousand- seed weight (18.9g), whereas seed rate of 15 kg ha ⁻ ¹ produced the lowest thousand seed weight (15.4g).

The combination of seed rate, inter-row spacing, and location also had a significant effect on grainyield, which ranged from 0.34 t ha ⁻ ¹ to 0.99 t ha ⁻ ¹. At Kete Kebele, the highest grain output (0.99 t ha ⁻ ¹) was recorded at 20 cm inter-row spacing with a seed rate of 30 kg ha ⁻ ¹; at Korke Kebele, the lowest grain yield (0.34 t ha ⁻ ¹) was noted at 40 cm inter-row spacing with a seed rate of 15 kg ha ⁻ ¹.

The interaction between seed rate and inter- row spacing had a significant (P ≤ 0.001) effect on grainyield. Inter-row spacing of 20 cm and a seed rate of 30 kg ha ⁻ ¹ produced the highest grain yield (0.98t ha^-1^), while inter-row spacing of 40 cm and a seed rate of 15 kg ha ⁻ ¹ produced the lowest grain yield (0.36t ha^-1^) (Table 4).

Grain yield was significantly (P ≤ 0.001) affected by location. Kete kebele had the highest grain yield (0.68 t ha^-1^), while Korke kebele had the lowest (0.66 t ha^-1^). Grain yield had a significant (P ≤ 0.001) effect on inter- row spacing. Inter-row spacing of 20 cm produced the highest grain yield (0.74 t ha^-1^), whereas inter-row spacing of 40 cm produced the lowest grain yield (0.61 t ha^-1^).

Grain yield had a significant (P ≤ 0.001) effect on seed rate. A seed rate of 30 kg ha ⁻ ¹ produced the highest grain yield (0.82 t ha^-1^), whereas a seed rate of 15 kg ha ⁻ ¹ produced the lowest grain yield (0.45 t ha^-1^).

#### Effect on biological yield and harvest index.

Biological yield was not significantly affected by the interaction of seed rate, inter-row spacing, and location.

The combination of seed rate and inter-row spacing had a significant effect on biological yield. Plants sown at 20 cm inter-row spacing with a seed rate of 30 kg ha ⁻ ¹ produced the highest biological yield (6.7t ha^-1^), whereas plants sown at 40 cm inter-row spacing with a seed rate of 15 kg ha ⁻ ¹ produced the lowest biological yield (3.4t ha^-1^) (Table 4).

Biological yield was significantly (P ≤ 0.001) affected by location. Korke kebele had the lowest biological yield weight (5 t ha^-1^), but Kete kebele had the highest biological yield (5.2 t ha^-1^). Biological yield had a significant (P ≤ 0.001) effect on inter- row spacing. Inter-row spacing of 20 cm produced the highest biological yield (5.6 t ha^-1^), whereas inter-row spacing of 30 cm produced the lowest biological yield (4.7 t ha^-1^). Biological yield had a considerable (P ≤ 0.001) impact on seed rate. A seed rate of 30 kg ha ⁻ ¹ produced the largest biological yield (5.8 t ha^-1^), whereas a seed rate of 15 kg ha ⁻ ¹ produced the lowest biological yield (3.9 t ha^-1^).

## 4. Discussions

### 4.1. Growth and phenological parameters as affected by seed rate and inter-row spacing of fenugreek

#### Effect on plant height.

The combined analysis across locations indicated that the interaction between seed rate and inter-row spacing did not significantly affect plant height, suggesting that these factors influenced vertical growth independently. However, inter-row spacing and seed rate each showed significant main effects on plant height. Plants grown at inter-row spacing of 40 cm attained the maximum height (49.70 cm), whereas those grown at 30 cm were shorter (46.96 cm). The increased plant height observed could be attributed to reduced inter-plant competition, which enhances access to light, soil moisture, and nutrients, thereby promoting vegetative growth.

Seed rate also significantly influenced plant height, increasing from 45.07 cm at 15 kg ha ⁻ ¹ to 49.80 cm at 35 kg ha ⁻ ¹. This response is likely associated with competition-induced stem elongation under denser plant populations, where plants grow taller to maximize light interception. Similar findings have been reported by [16–18] who observed increased plant height at higher planting densities or seed rates in fenugreek and other legumes. Likewise, [19] reported taller faba bean plants under higher plant density, attributing the response to increased competition for light and other growth resources.

In contrast, [20] reported that planting density had no significant effect on plant height under certain growing conditions. Such inconsistencies among studies suggest that the influence of planting density on plant height is not universal but depends on species, genotype, environmental conditions, soil fertility, and crop management practices.

#### Effect on number of branches per plant.

The number of branches per plant was significantly affected by the interaction of spacing, seed rate, and location, indicating that the response of branching to planting geometry varied across environments. The higher number of branches observed under favorable combinations of inter-row spacing and seed rate could be associated with reduced inter-plant competition and improved availability of light, soil moisture, and nutrients. Wider row spacing also enhances canopy light penetration, which promotes vegetative growth and the development of lateral branches. Similar findings have been reported by [21–23] who observed that increasing row spacing significantly increased the number of branches per plant in mung bean, common bean, and cumin, respectively. These results suggest that the branching response to plant density is largely determined by the balance between resource availability and competition, with the magnitude of the response depending on both environmental conditions and crop management practices.

#### Effect on days to 50% flowering.

The delayed flowering observed at higher seed rates could be attributed to increased competition among plants under dense populations, which reduces the availability of light, nutrients, and soil moisture to individual plants. Such competition can slow vegetative development and delay the transition from vegetative to reproductive growth, resulting in a longer time to reach 50% flowering. Differences in flowering time among locations may also reflect variations in environmental conditions, particularly temperature and soil moisture, which influence crop phenology. These findings are consistent with those of [9], who likewise reported delayed flowering at higher plant densities. Overall, the results indicate that both plant population density and environmental conditions play important roles in regulating flowering time in fenugreek.

#### Effect on 90% physiological maturity.

Days to 90% physiological maturity were significantly affected by location, inter-row spacing, and seed rate independently. Favorable environments often prolong the grain filling period, resulting in delayed maturity. Wider spacing reduces competition among plants, allowing prolonged vegetative growth and extended grain filling duration by improving access to resources. In contrast, closer spacing intensifies competition, accelerating crop senescence and shortening the crop cycle. Higher plant density increases competition and canopy shading, which can slow down assimilate partitioning and extend the reproductive phase. Conversely, lower density enhances resource availability per plant, leading to faster development and earlier completion of the crop cycle. These results are consistent with the findings of [9,24], who noted that higher plant density caused delayed flowering and maturity. In fenugreek, the earliest blooming was observed at the lowest seed rate of 16 kg ha^-1^ and occurred 6.41 days earlier in 50% of plants [17]. The current results, however, are inconsistent to those of [25], who found that phenological development responded differently to diverse plant densities.

### 4.2. Nodulation, Yield Components and Yield Parameters as Affected by Seed Rate and Inter-Row Spacing of Fenugreek

#### Effect on number of nodules per plant.

The significant interaction between seed rate and inter-row spacing indicates that nodulation in fenugreek depends on the combined influence of plant population and spatial arrangement. The highest number of nodules was obtained at a 30 kg ha ⁻ ¹ seed rate with 20 cm inter-row spacing, suggesting that this combination provided favorable conditions for root development and root–rhizobia interactions, thereby enhancing nodule formation. In contrast, the lowest nodulation at 15 kg ha ⁻ ¹ with the same inter-row spacing may reflect reduced root density and fewer opportunities for effective rhizobial infection. Similar responses have been reported in legumes, where plant population and crop establishment practices influence nodulation and biological nitrogen fixation [26,27].

The significant main effects of location, inter-row spacing, and seed rate further indicate that environmental conditions and crop management collectively regulate nodulation. The higher number of nodules recorded at Kete compared with Korke is likely associated with differences in soil properties and native rhizobial populations, which influence rhizobial survival, root infection, and nodule development [6,27,28]. Overall, these findings suggest that adopting an appropriate combination of seed rate and row spacing under favorable site conditions can enhance nodulation and potentially improve biological nitrogen fixation in fenugreek.

#### Effect on number of pods per plant and number of seeds per pod.

The significant interaction between seed rate and inter-row spacing indicates that the combined effects of planting density and crop geometry influenced reproductive development in fenugreek. In the present study, the greatest number of pods per plant and seeds per pod was obtained at a inter-row spacing of 20 cm combined with a seed rate of 30 kg ha ⁻ ¹, suggesting that this treatment provided a favorable balance between plant population and resource availability. This combination may have promoted efficient canopy development and assimilate production while minimizing excessive competition among plants, thereby enhancing reproductive growth. These findings are generally consistent with those of [17,29,30] who reported that appropriate combinations of row spacing and seed rate increased the number of pods per plant in fenugreek. However, [16] observed a decline in yield-related traits with increasing row spacing, supporting the present results that excessively wide spacing may reduce reproductive performance. In contrast, [11] reported a negative relationship between seed rate and the number of pods per plant. Such discrepancies among studies may be attributed to differences in environmental conditions, soil fertility, cultivar characteristics, and crop management practices, all of which influence how reproductive traits respond to planting density.

#### Effect on thousand-seed weight and grain yield.

The interaction between seed rate and inter-row spacing significantly influenced thousand-seed weight and grain yield of fenugreek, indicating that an appropriate balance between plant population and spatial arrangement is important for optimizing reproductive performance. The increase in thousand-seed weight under the optimum treatment combination may be associated with improved assimilate availability during seed development and more efficient partitioning of photosynthates toward developing seeds. However, excessive competition under very high plant density may reduce seed filling by limiting the availability of growth resources.

The highest grain yield (0.99 t ha ⁻ ¹) was recorded at a inter-row spacing of 20 cm combined with a seed rate of 30 kg ha ⁻ ¹. This result suggests that the optimum plant density achieved under this treatment enhanced yield formation through increased plant population per unit area while maintaining adequate access to essential resources. Narrower row spacing promotes faster canopy development, improved light interception, and greater biomass accumulation, which ultimately contributes to higher seed yield. Conversely, the lowest grain yield observed under the wider inter-row spacing (40 cm) and lower seed rate (15 kg ha ⁻ ¹) could be attributed to insufficient plant population and reduced utilization of available growing resources, resulting in fewer reproductive units per unit area.

Similar results have been reported by [21,31], who observed increased seed yield with increasing plant population, while [24] indicated that sparse plant populations reduce resource capture and limit yield potential. The slightly higher yield observed at Kete compared with Korke may be related to differences in environmental conditions, including rainfall distribution, soil fertility, and other site-specific factors that influence crop growth and yield formation.

The results demonstrate that optimizing seed rate and row spacing is essential for maximizing fenugreek productivity, as inappropriate planting density can either increase competition among plants or reduce the efficient utilization of available resources.

#### Effect on biological yield and harvest index.

The interaction between seed rate and inter-row spacing significantly influenced biological yield, with the highest biomass yield recorded at inter-row spacing of 20 cm combined with a seed rate of 30 kg ha ⁻ ¹. The greater biological yield under this treatment could be attributed to increased plant population per unit area and improved canopy development, resulting in enhanced light interception and greater dry matter accumulation. At the optimum plant density, narrow row spacing facilitates more effective capture of available growth resources, including light, water, and nutrients, thereby promoting biomass production.

In contrast, the lowest biological yield observed under wider inter-row spacing and lower seed rate was likely associated with reduced plant population and incomplete canopy development, which limited resource capture and biomass accumulation. Although increased plant density may reduce the growth of individual plants due to competition, the greater number of plants per unit area generally results in higher total biomass production. Similar findings were reported by [17,32,33], who observed increased dry matter yield with increasing planting density.

The higher biological yield recorded at Kete compared with Korke could be related to differences in environmental conditions, including rainfall distribution, soil properties, and other site-specific factors that influence vegetative growth. Regarding harvest index, the variation among treatments reflects differences in the efficiency of assimilate partitioning from vegetative biomass to grain production. The higher harvest index observed under the optimum seed rate and inter-row spacing combination indicates improved reproductive efficiency and better allocation of photosynthates toward seed formation.

These findings highlight the importance of optimizing seed rate and row spacing to balance biomass production and assimilate partitioning, thereby maximizing overall fenugreek productivity.

## 5. Conclusion

This study demonstrated that inter-row spacing and seed rate significantly influenced the growth, nodulation, dry matter accumulation, yield components, and grain yield of fenugreek under the agroecological conditions of the Tehuledere District, northeastern Ethiopia. The significant interaction between these two agronomic factors highlights the importance of optimizing planting geometry and plant population density to improve fenugreek productivity.

Among the evaluated treatments, the combination of 20 cm inter-row spacing and 30 kg ha ⁻ ¹ seed rate consistently resulted in superior crop performance, producing the highest grain yield (0.98 t ha ⁻ ¹) through improved yield components, effective biomass production, and better utilization of available growth resources. Therefore, 20 cm inter-row spacing combined with a seed rate of 30 kg ha ⁻ ¹ can be recommended as an appropriate agronomic practice for fenugreek production in Tehuledere District and similar agroecological environments.

However, considering that the study was conducted at two locations during a single growing season, further multi-season and multi-location studies are suggested to validate the stability, wider applicability, and long-term economic performance of this recommendation under diverse environmental conditions.

## Supporting information

S1 File sr zenodo. (DOCX)

## References

[1] Kumar S, Sharma V, Verma AK, Bhardwaj V. Fenugreek genetic resources. Vegetable Crops. Singapore: Springer. 2025. p. 1287–316.

[2] Ahari DS, Kashi AK, Hassandokht MR, Amri A, Alizadeh K. Assessment of drought tolerance in Iranian fenugreek landraces. J Food Agric Environ. 2009;7:414–9.

[3] Bekele D, Lulseged T, Yadeta D, Kifelew H, Getu A, Demis F, et al. Release of fenugreek (Trigonella foenum-graecum L.) variety Bishoftu. Afr J Agric Res. 2020;16(12):1726–9.

[4] Kifle H, Fikere D, Lulseged T, Bekele D, Mitiku H, Getachew W. Seed spices production guideline. Addis Ababa, Ethiopia: Ethiopian Institute of Agricultural Research. 2017.

[5] Shadab M, Akhtar N, Quratul-ain, Siddiqui MB. Medicinal and Nutritional Importance of Trigonella foenum-graecum in Human Health. Medicinal Plants and their Bioactive Compounds in Human Health: Volume 1. Springer Nature Singapore. 2024. p. 123–41. doi: 10.1007/978-981-97-6895-0_7

[6] Liu A, Contador CA, Fan K, Lam HM. Interaction and regulation of nodulation in legumes. Frontiers in Plant Science. 2013;4:1. 30619423 10.3389/fpls.2018.01860 PMC6305480

[7] David B. Globalization and the developing countries: Emerging strategies for rural development and poverty alleviation. Wallingford: CABI Publishing. 2002.

[8] Kakani RK, Saxena SN, Meena SS, Chandra P. Stability analysis for yield and yield attributes in fenugreek under water limiting conditions. Int J Seed Spices. 2014;4(2):47–52.

[9] Kumar P, Phor SK, Mor VS, Kumar S, Mittal S. Study the effect of seed rate and row spacing on quality parameters of fenugreek (Trigonella foenum-graecum L.). Int J Chem Stud. 2018;6(4):454–6.

[10] Tiwari D, Upadhyay S, Paliwal A. Plant spacing response on growth and yield of fenugreek in high altitude of Uttarakhand. Int J New Technol Res. 2016;2(10):33–5.

[11] Tunçtürk R. The effects of varying row spacing and phosphorus doses on the yield and quality of fenugreek (Trigonella foenum-graecum L.). Turk J Field Crops. 2011;16(2):142–8.

[12] Central Statistical Agency CSA. Agricultural sample census survey: Crop and livestock production. Addis Ababa, Ethiopia: CSA. 2021.

[13] Tesfa T, Bayu W, Gashaw A, Beshir H. Spice production, marketing, and utilization in South Wollo, Ethiopia. East Afr J Sci. 2017;11(1):27–36.

[14] Jari Agricultural Research Sub Center. Annual report. Jari, Ethiopia; 2015.

[15] Gutema C. Growth, yield, and seed protein content of fenugreek (Trigonella foenum-graecum L.) varieties as influenced by application of NPS fertilizer at Ginir, southeastern Ethiopia. 2021.

[16] Singh D, Sani SS, Salaria A, Gill BS. Agronomic manipulations for maximum production of Trigonella foenum-graecum L. in Punjab. Indian J Arecanut Spices Med Plants. 2005;7(2):69–71.

[17] Kumar P, Phor SK, Tehlan SK, Mathur AK. Effect of seed rate and row spacing on growth and yield of fenugreek (Trigonella foenum-graecum L.). J Pharmacogn Phytochem. 2018;7(4):93–6.

[18] Tafesse M, Desalegn N, Adugna H. Influence of seed rate and row spacing on growth, yield and yield components of fenugreek (Trigonella foenum-graecum L.) in Abay Chomen District, western Ethiopia. J Agric Food Nat Resour. 2023;1(1):22–8.

[19] Roussis I, Travlos I, Bilalis D, Kakabouki I. Influence of seed rate and fertilization on yield and yield components of nigella sativa l. cultivated under mediterranean semi-arid conditions. AGROLIFE. 2017;6(1):218–23. doi: 10.17930/agl2017130

[20] Seghatoleslami MJ, Ahmadi Bonakdar K. The effect of sowing date and plant density on yield and yield components of fenugreek (Trigonella foenum-graecum L.). J Med Aromat Plants. 2009;26:265–74.

[21] Beze K, Shumbulo A. Optimization of NPS fertilizer rate and row spacing for growth and yield of fenugreek (Trigonella foenum-graecum L.) at Wolaita Sodo, southern Ethiopia. Agric Sci Dig. 2024. doi: 10.18805/ag.DF-578

[22] Gemechu E, Solomon T. Effect of NPS fertilizer rate and intra-row spacing on growth and yield of common bean (Phaseolus vulgaris L.) at Metu, southwestern Ethiopia. Int J Agric Innov Res. 2021;10(2).

[23] Kizil S, Kirici S, Sonmez O. Effect of different row distance on some agronomical characteristics and essential oil composition of cumin (Cuminum cyminum L.). Die Bodenkultur. 2008;59(1–4).

[24] Bohloul A, Fatemeh S, Masoumeh LH, Elaheh KH. The effect of planting time and planting density on yield and essential oil of Satureja sahendica Bornm. J Med Plants By-prod. 2014;:141–2.

[25] Pandita VK, Randhawa KS. Row spacing and leaf cutting in relation to seed production of fenugreek (Trigonella corniculata L.) cv. Pusa Kasuri. Seed Res. 1994;22(2):127–9.

[26] Schwember AR, Schulze J, Del Pozo A, Cabeza RA. Regulation of symbiotic nitrogen fixation in legume root nodules. Plants (Basel). 2019;8(9):333. doi: 10.3390/plants8090333 31489914 PMC6784058

[27] Yeremko L, Czopek K, Staniak M, Marenych M, Hanhur V. Role of environmental factors in legume-rhizobium symbiosis: a review. Biomolecules. 2025;15(1):118. doi: 10.3390/biom15010118 39858512 PMC11764364

[28] Zahran HH. Rhizobium–legume symbiosis and nitrogen fixation under severe conditions and in an arid climate. American Soc Microbiol. 1999. p. 968–89. 10.1128/mmbr.63.4.968-989.1999 PMC98982 10585971

[29] Yadav JS, Singh J, Kumar V, Yadav BD. Effect of sowing time, spacing and seed rate on seed yield of fenugreek (Trigonella foenum-graecum L.) in light textured soil. HAU J Res. 2000;30(3–4):107–11.

[30] Meena BR, Jat NL, Meena RP. Varietal response of fenugreek (Trigonella foenum-graecum L.) to row spacing and phosphorus nutrition in relation to growth attributes and yield. Ann Agric Biol Res. 2003;8(2):201–4.

[31] Ghahari M, Reza B, Reza F. Effects of planting density and weeding time on weeds and fenugreek dry matter. Middle East J Sci Res. 2013;18(8):1191–8.

[32] Nandal JK, Dahiya MS, Gupta V, Singh D. Response of sowing time, spacing and cutting of leaves on growth and seed yield of fenugreek. Haryana J Hortic Sci. 2007;36(3–4):374–6.

[33] Naghdi Badi H, Yazdani D, Ali SM, Nazari F. Effects of spacing and harvesting time on herbage yield and quality/quantity of oil in thyme, Thymus vulgaris L. Industrial Crops and Products. 2004;19(3):231–6. doi: 10.1016/j.indcrop.2003.10.005

