# Supplementary material for: Effect of inter- row spacing and seed rate on growth; Yield and yield components of fenugreek (Trigonella foenum - graecum L.) in Tehuledere district, Northeastern Ethiopia
Source: PLoS One. 2026 Aug 14;21(8):e0355598. doi: 10.1371/journal.pone.0355598 (PMC13475882; doi:10.1371/journal.pone.0355598)
Supplement: Supplementary file 1 [file pone.0355598.s001.docx]

<https://doi.org/10.5281/zenodo.19682010>
